# Microstructural abnormalities of substantia nigra in Parkinson's disease: A neuromelanin sensitive MRI atlas based study

**DOI:** 10.1002/hbm.24878

**Published:** 2019-11-28

**Authors:** Apoorva Safai, Shweta Prasad, Tanay Chougule, Jitender Saini, Pramod K. Pal, Madhura Ingalhalikar

**Affiliations:** ^1^ Symbiosis Center for Medical Image Analysis, Symbiosis Institute of Technology Symbiosis International University Pune Maharashtra India; ^2^ Department of Clinical Neurosciences National Institute of Mental Health & Neurosciences Bangalore Karnataka India; ^3^ Department of Neurology National Institute of Mental Health & Neurosciences Bangalore Karnataka India; ^4^ Department of Neuroimaging & Interventional Radiology National Institute of Mental Health & Neurosciences Bangalore Karnataka India

**Keywords:** atlas, diffusion tensor imaging, Neuromelanin, Parkinson's disease, substantia Nigra

## Abstract

Microstructural changes associated with degeneration of dopaminergic neurons of the substantia nigra pars compacta (SNc) in Parkinson's disease (PD) have been studied using Diffusion Tensor Imaging (DTI). However, these studies show inconsistent results, mainly due to methodological variations in delineation of SNc. To mitigate this, our work aims to construct a probabilistic atlas of SNc based on a 3D Neuromelanin Sensitive MRI (NMS‐MRI) sequence and demonstrate its applicability to investigate microstructural changes on a large dataset of PD. Using manual segmentation and deformable registration we created a novel SNc atlas in the MNI space using NMS‐MRI sequences of 27 healthy controls (HC). We first quantitatively evaluated this atlas and then employed it to investigate the micro‐structural abnormalities in SNc using diffusion MRI from 133 patients with PD and 99 HCs. Our results demonstrated significant increase in diffusivity with no changes in anisotropy. In addition, we also observed an asymmetry of the diffusion metrics with a higher diffusivity and lower anisotropy in the left SNc than the right. Finally, a multivariate classifier based on SNc diffusion features could delineate patients with PD with an average accuracy of 71.7%. Overall, from this work we establish a normative baseline for the SNc region of interest using NMS‐MRI while the application on PD data emphasizes on the contribution of diffusivity measures rather than anisotropy of white matter in PD.

## 1 INTRODUCTION

Parkinson's disease (PD) is a chronic, progressive disorder typically characterized by bradykinesia, rigidity, and tremor, and these symptoms have been implicated to degeneration of dopaminergic neurons in the substantia nigra pars compacta (SNc). These dopaminergic neurons contain neuromelanin, loss of which manifests as depigmentation of the SNc. This has been well established as an early histological feature of PD (Fearnley & Lees, 1991). Degeneration of these nigral, dopaminergic neurons may lead to alterations in microstructural organization of the regional gray matter, white matter and local myelination of the SNc in PD.

To gain understanding of the underlying microstructural changes in the SNc, studies have relied upon anisotropy and diffusivity measures computed from diffusion tensor magnetic resonance imaging (DT‐MRI or DTI). Typically, these studies initially delineate the SNc and then perform analysis on the computed ROI. Table 1 provides a brief review of existing studies that provides the reported DTI findings in PD and is classified based on the technique used for SNc localization. Majority of these studies (Chan et al., 2007; Du et al., 2011; Knossalla et al., 2018; Langley et al., 2016; Loane et al., 2016; Peran et al., 2010; Rolheiser et al., 2011; Schwarz et al., 2013; Vaillancourt et al., 2009; Zhan et al., 2012) demonstrate a significant difference between PD and HC groups in at least one of the diffusion measures of SNc such as fractional anisotropy (FA), mean diffusivity (MD), radial diffusivity (RD) and axial diffusivity (AD). Contradictory to these findings, some studies (Aquino et al., 2014; Gattellaro et al., 2009; Menke et al., 2010; Schuff, 2015) report no significant changes in any of these diffusion measures in PD.

**Table 1:** Review of articles that have performed diffusion MRI analysis on the substantia nigra pars compacta in Parkinson's disease

Author (year)	Sample size (DTI/NM‐MRI)	ROI placement	ROI image	DTI results of PD in comparison to HC
***DTI studies with T1/Neuromelanin based SN contrast*** **:**				
Langley et al. (2016)	20:17/11 (PD: HC /HC)	Manual	NM‐T1, T2w	FA↓, MD↑ for NM‐T1
Menke, Jbabdi, Miller, Matthews, and Zarei (2010)	10:10 (PD: HC)	Semiautomatic	T1w, DTI tracks	No significance
DTI studies with T2w/ T2*w image based SN contrast **:**				
Schwarz et al. (2013)	32:27 (PD: HC)	Manual	T2w	MD↑
Du et al. (2011)	16:16 (PD: HC)	Semiautomatic	T2w	FA↓, R2*↑
Prakash et.al (2012)	11:12 (PD: HC)	Manual	T2w	FA asymmetry in SNc subregion in PD
Rolheiser et al. (2011)	14:14 (PD: HC)	Manual	T2w + V1 maps	FA↓, RD↑
Peran et al. (2010)	30:22 (PD: HC)	Manual	T2*w images	FA↓, R2*↑
Vaillancourt et al. (2009)	14:14 (PD: HC)	Manual	T2w	FA↓
***DTI studies with DTI based SN contrast*** **:**				
Knossalla et al. (2018)	10:10 (PD: HC)	Manual	FA maps	FA↓
				Asymmetry in SNc subregion
Loane et al. (2016)	18:14 (PD:HC)	Manual	FA, MD maps	FA↓, MD↑
Schuff et al (2015)	67:153 (PD:HC)	Manual	FA maps, T1w	FA↓ in rostral SNc, AD↑, RD
				No significance
Aquino et al. (2014)	42:20 (PD:HC)	Manual	Axial IR	No significance
Zhan et al. (2012)	12:20 (PD:HC)	Semiautomatic	FA maps	FA↓
Gattellaro et al. (2009)	10:10 (PD:HC)	Manual	DTI tracks	No significance
Chan et al. (2007)	73:78 (PD:HC)	Manual	FA maps	FA↓

Abbreviations: ↑: Increase; ↓: Decrease; DTI: Diffusion Tensor Imaging; FA: Fractional Anisotropy; HC: Healthy Controls; MD: Mean Diffusivity; NM‐MRI: Neuromelanin Magnetic Resonance Imaging; NM‐T1: Neuromelanin sensitive T1; PD: Parkinson's disease; RD: Radial Diffusivity; ROI‐Region Of Interest; SNc: Substantia Nigra pars compacta; T1w: T1 weighted; T2w: T2 weighted.

Taken together, the findings have been widely heterogeneous and perhaps could be implicated to inconsistencies in stages of disease severity as well as to variable sample sizes. More importantly, the techniques employed for delineating the SNc may have a significant bearing on the variability in the reported results. For example, studies have employed T2 weighted, proton density weighted spin echo and inversion recovery based contrasts to precisely localize the SNc ROIs (Atasoy et al., 2004; Duguid, De La Paz, & DeGroot, 1986; Geng, Li, & Zee, 2006; Hutchinson & Raff, 2000; Oikawa, Sasaki, Tamakawa, Ehara, & Tohyama, 2002; Pujol, Junque, Vendrell, Grau, & Capdevila, 1992; Stern, Braffman, Skolnick, Hurtig, & Grossman, 1989; Tuite, Mangia, & Michaeli, 2013). However, in a conventional T2 weighted MRI, there is variability in the hypo‐intensity associated with the SNc as a result of increased iron deposition, in addition to the reduction in neuromelanin content (Deng, Wang, Yang, Li, & Yu, 2018; Langley et al., 2015; Langley et al., 2016; Wypijewska et al., 2010). This often leads to an inaccurate marking of SNc boundaries and a false pathological representation of dopamine degeneration (Langley et al., 2016). To obtain more precision in SNc delineation, recent studies have relied upon more sophisticated MRI protocols such as DTI where fiber tracking is employed to extract the SNc. (Menke et al., 2010; Sasaki et al., 2006; Zhang et al., 2015). However, this technique is highly dependent upon the choice of diffusion MRI protocol and the fiber tracking algorithm as well as is susceptible variations in manual fiber tracking. Nonetheless, direct visualization and segmentation of the SNc, is therefore, a simpler yet a precise option to ensure superior accuracy in analysis of PD. To this end, a novel MR sequence known as “neuromelanin‐sensitive MRI” (NMS‐MRI) has demonstrated encouraging results.

NMS‐MRI which is a 3 T T1‐weighted high‐resolution fast spin‐echo sequence is highly sensitive to the neuromelanin contained in the SNc and therefore renders the SNc as a hyper intense structure (Sasaki et al., 2006; Sasaki et al., 2008). This sequence is based on the paramagnetic properties of neuromelanin, a neuronal pigment which is a by‐product of dopamine synthesis. Owing to the dopaminergic neuron loss in patients with PD, this normally hyper intense structure shows loss of normal signal intensity on NMS‐MRI and therefore can be considered as a biomarker for PD. Multiple studies have demonstrated the clinical utility and accuracy of this sequence in patients with PD (Castellanos et al., 2015; Ohtsuka et al., 2013; Ohtsuka et al., 2014; Schwarz et al., 2011). To quantify these differences, the processing techniques that are currently used are based on visual inspection, or manual region of interest drawing followed by computation of volumes, contrast ratios or radiomics features and are arduous and time‐consuming (Isaias et al., 2016; Kashihara, Shinya, & Higaki, 2011; Matsuura et al., 2013; Matsuura et al., 2016; Ogisu et al., 2013; Ohtsuka et al., 2014; Reimao et al., 2015; Sasaki et al., 2006; Schwarz et al., 2011; Shinde et al., 2019). To overcome this, NMS‐MRI sequence can be employed to accurately localize and create a template of the SNc that can be utilized for analysis in parkinsonian disorders. Delineating the SNc in healthy controls and creating a template will not only offer better anatomical context to future studies but also provide a normative baseline and a ground‐truth for comparison of multiple populations.

To this end, our work aims to generate a probabilistic atlas of the SNc from NMS‐MRI and endeavors to apply the atlas on a large group of patients with PD to gain deeper insights into the diffusion MRI based microstructural abnormalities of the SNc. The generated probabilistic atlas of the SNc will provide a simple method for localization of the SNc, mitigating the methodological lack of uniformity in future studies.

## 2 METHODS

### 2.1 Subject recruitment and clinical evaluation

A total of 133 subjects with PD and 99 healthy controls were recruited from two studies that were conducted at the Department of Neurology, National Institute of Mental Health and Neurosciences (NIMHANS), Bangalore, India. The diagnosis of idiopathic PD was based on the UK Parkinson's Disease Society Brain Bank criteria (Hughes, Daniel, Kilford, & Lees, 1992) and confirmed by a trained movement disorder specialist (author PKP). Patients included in this study have been part of other studies (Lenka et al., 2018; Shah et al., 2017) from this group and all patients and controls provided informed consent prior to recruitment in the original projects. Data usage for this study was reviewed and approved by the review board at NIMHANS.

Demographic and clinical details such as gender, age at presentation, age at onset of motor symptoms, disease duration, Mini Mental State Examination (MMSE), and Unified Parkinson's Disease Rating Scale (UPDRS‐III) OFF‐state scores, and levodopa equivalent daily dose (LEDD) were recorded. The OFF state was defined as 12 hr after the last dose of levodopa, and 48 hr after the last dose of a dopamine agonist. Age and gender matched healthy controls with no family history of Parkinsonism or other movement disorders were recruited.

Another group of 27 healthy controls (Age = 38.67 ± 11.01, gender [M:F] =18/9) whose NMS‐MRI sequence was acquired as part of a different study (Prasad et al., 2018) of the same group, was used in the construction of our probabilistic atlas.

### 2.2 Image acquisition

All subjects were scanned on a 3 T Philips Achieva MRI scanner using a 32‐channel head coil. Diffusion weighted images (DWI) for these subjects were acquired using a single‐shot spin‐echo EPI sequence with repetition time (TR) = 8,583–9,070 ms, echo time (TE) = 60‐62 ms, field of view (FOV) = 128 × 128 × 70m, voxel size = 1.75 × 1.75 × 2mm, slice thickness = 2 mm. Diffusion gradient was applied in 15 directions, with *b* value =1000 s/mm and a single *b* = 0 s/mm. T1‐weighted images were acquired using TR = 8.06 ms, TE = 3.6 ms, voxel‐size = 1 × 1 × 1mm, FOV = 256 × 256 × 160mm, slice thickness = 1 mm, voxel size = 1 × 1 × 1mm and flip angle = 8.

For creating a probabilistic atlas of SNc, a different set of controls were scanned on the same scanner. T1‐weighted images were acquired using the above‐mentioned protocol. Neuromelanin contrast sensitive sequences were acquired using a fast spin echo 3D T1 acquisition with TE: 2.2 ms, TR: 26 ms; flip angle: 20°; reconstructed matrix size: 512 × 512; field of view: 180 × 180 × 50mm; voxel size: 0.9 × 0.9 × 1mm; number of slices: 50; slice thickness: 1 mm; and acquisition time: 4 min 12.9 s. These images covered only the areas between the posterior commissure and inferior region of pons.

### 2.3 Atlas construction

**Figure 1:**
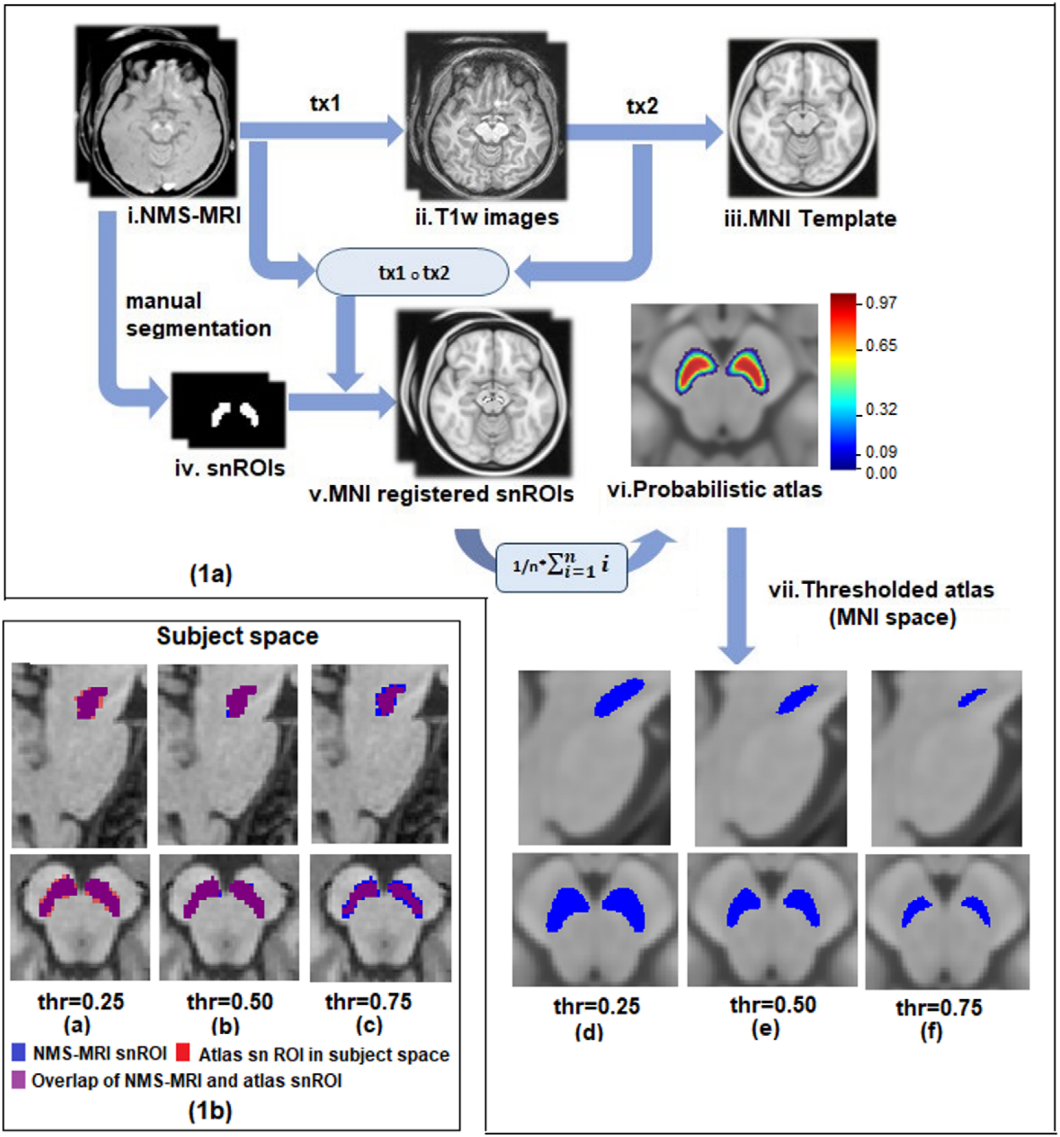
(a) Schematic for probabilistic atlas construction of substantia nigra in MNI space with thresholding. i: ith subject; n: number of subjects; tx1: transformation matrix of NMS‐MRI to T1 registration, tx2: transformation matrix of T1 to MNI registration. (b) Probabilistic and thresholded substantia nigra atlas in subject space

Probabilistic atlas was built from NMS‐MRI images of 27 subjects as shown in Figure 1. For each subject, bilateral substantia nigra ROIs (snROIs) were created from NMS‐MRI scans by manual segmentation. An author with expertise in the NMS‐MRI sequence (rater1 [R1]‐author SP), delineated the right and left SNc on the axial slices and created a 3D binary mask (snROIs) for all 27 subjects. The NMS‐MRI images were then linearly registered to the T1 image of the same subject by performing affine transformation using FLIRT in FSL (Smith et al., 2004). The T1 images of all subjects were preprocessed by performing motion correction, intensity inhomogeneity correction and skull stripping using Freesurfer 6.0 (Fischl, 2012) and were transformed to the MNI space, by employing a deformable registration using Advanced Normalization Tools (ANTS), wherein a symmetric diffeomorphic transformation model (SyN) was applied and optimized using mutual information. The SyN is a large deformation registration algorithm, which performs a bidirectional diffeomorphism and regularization using Gaussian smoothing of the velocity fields and has shown to outperform other nonlinear registration algorithms in preserving brain topology (Avants, Epstein, Grossman, & Gee, 2008). The transformations from NMS‐MRI to T1 and from T1 to MNI were concatenated and were applied to the snROIs to transform them to MNI space. Along with visual inspection of each registered image, mean and variance of registered images was computed for quality check of registration. A probabilistic atlas of SNc in MNI space was then created, such that the voxel contained in all the 27 snROIs, was labeled with a probability of 1 and voxels not contained in any of the 27 ROIs were labeled with a 0 probability. For all further analysis, the atlas was thresholded at probability of 0.5 in order to largely accommodate all voxel and to avoid over estimation of SNc in the atlas and as shown in Figure 1b.

### 2.4 Quantitative validation of atlas

Two additional sets of snROI markings were generated, one was by manual segmentation (by rater 2 [R2]‐author A.S) and the other was generated by a fully automated deep learning model known as U‐Net (Ronneberger, P, & Brox, 2015) was used for segmentation of SNc. Out of the dataset of 27 NMS‐MRI images, 10 were used as training dataset and 17 were used for testing. A 128 × 128 × 4 sized patch of the NMS‐MRI image was given as input to the model and their corresponding manually extracted SNc ROIs were used as target labels for segmentation (considered as rater 3 [R3]).

Dice coefficient, a standard measure of validation used in various atlas construction studies (Ariz et al., 2018; Pauli, Nili, & Tyszka, 2018; Visser et al., 2016) was computed to assess inter‐rater variability in SNc markings and validity of the constructed atlas. For R3, dice score with other raters and atlas was computed on the testing data with an exclusion of two data points that were outliers. Initially dice coefficient was calculated between the three raters to measure the amount of variability between SNc markings from different raters. To quantitatively validate the atlas, dice coefficient was computed between SNc atlas in subject space and snROIs from all three raters. The template SNc was brought into the subject space using an inverse transformation of the concatenated NMS‐MRI to T1 and T1 to MNI deformations. The registration details have been provided in the earlier Section 2.3.

To further investigate whether the overlap amongst SNc markings significantly differs from the overlap of atlas and raters markings, we evaluated the statistical significance by testing for the means of dice coefficient scores of raters (R2 and R3) and combined dice scores of atlas with raters R2 and R3 using a Student's *t* test.

### 2.5 DTI preprocessing and analysis

DWI images of patients with PD and healthy controls were manually visualized for quality assessment. All preprocessing steps were done using FSL5.0.9 (Smith et al., 2004) which included removing the nonbrain regions, correction for head movement and eddy current induced distortions using “eddy correct” tool that performs an affine transformation between baseline b0 image and gradient images. The resulting rotating parameters of the affine transformation were used to rotate the gradients back, to align them with the transformed images. Least square approximation method was implemented to reconstruct the diffusion tensor images using “dtifit”, and the tensor fitting was checked for anatomical alignment. Diffusion maps such as fractional anisotropy (FA), mean diffusivity (MD), radial diffusivity (RD) and axial diffusivity (AD) were obtained by fitting the diffusion tensor model. FA maps of all subjects were registered to a standard FA map in MNI space‐FMRIB58 image (https://fsl.fmrib.ox.ac.uk/fsl/fslwiki/FMRIB58_FA) using SyN algorithm and mutual information similarity index in ANTS. The transformation matrix of this registration was used to transform the MD, RD, and AD maps to MNI space. Diffusion measures of bilateral SNc were extracted for all subjects using MNI registered diffusion maps and the atlas described in earlier, which was thresholded at a 0.5 probability.

### 2.6 Statistical analysis

Statistical analysis on diffusion measures between PD and HC was performed using multivariate analysis of covariance (MANCOVA) model, with FA, MD, RD, AD of left, and right SNc as dependent variables, PD and HC as grouping variables and age and gender as covariates. In addition, an independent *t* test was performed between diffusion measures of HC and PD groups. The asymmetric pathological nature of PD is a key feature which aids in differentiating it from atypical parkinsonian disorders. In order to ascertain if this asymmetry is also reflection in diffusion metrics, an independent *t* test was implemented between DTI measures of left and right SNc for PD patients and separately for HC. All these t‐tests were corrected for multiple comparisons using false discovery rates (FDR) threshold of 0.05.

To evaluate associations between the microstructural changes to the severity of the disease, the residuals from the diffusion measures after regressing out age and gender were correlated to the UPDRS‐III OFF scores (where available), the age of onset of disease (AoO), duration of illness (DoI) and LEDD of patients with PD.

### 2.7 Classifier for differentiating PD patients from HC

Further, to understand the discriminative power of these diffusion measures, a multivariate random forest (RF) was implemented to delineate PD from the HC. RF is a popular decision tree based machine‐learning algorithm (Breiman, 2001). At each node of a tree, different subset of randomly selected predictors are considered, of which the best predictor is selected for further splits. Each tree is built using a different random bootstrap sample, which consists of approximately two‐thirds of the total observations.

In this study, RF model was implemented on a total dataset of 232 subjects, out of which 75% was used for training and 25% for testing. A total of eight normalized diffusion measures comprising AD, FA, MD, RD for left and right SNc were used as feature set for the classifier.

Number of predictors or features sampled for splitting at each node and the number of trees in the forest were the two primary tuning parameters in the model (Liaw & Wiener, 2002). The RF model was tuned on the following parameters—number of trees (between 500 to 1,500), number of samples at each leaf (1 to maximum samples) and number of samples at each split (2 to maximum samples). Gini impurity was used as the criterion for selecting the best split at each node. Features were ranked based on their feature importance. Further, in order to eliminate any possible bias in the obtained feature rankings due to correlated diffusion features, a RF model using Recursive Feature Elimination (RF‐RFE) with Cross Validation was also implemented on the training dataset. The RFE model was iteratively trained, wherein the first run was initiated from the basic RF model, followed by (a) recursive elimination of features with lowest importance score until only one feature is left. (b) At every step, fivefold cross validation was performed on the training data and the average accuracy across all folds was noted. (c) At each step, features were ranked based on the order in which they were removed along with their relative feature importance. This process was repeated 10 times to ensure stability in classification performance. The average accuracy, sensitivity and specificity and feature ranking from all repetitions were used to evaluate the model performance.

## 3 RESULTS

### 3.1 Demographic and clinical details

Table 2 provides the complete demographic and clinical information for the dataset under consideration. There were no differences observed between the age and gender of the patient and control group. UPDRS‐III OFF scores were available for 110 out of 135 subjects. With the exception of a single subject of PD, all others were right‐handed. The mean duration of illness was 5.08 ± 3.11, with a UPDRS III OFF score of 34.68 ± 8.80, and LEDD of 598.39 ± 238.50.

**Table 2:** Demographic and clinical characteristics of patients with Parkinson's disease and healthy controls

	Parkinson's disease (*n* = 133)	Healthy controls (*n* = 99)	*p* value
Gender (M: F)	104:29	71:28	0.257
Age	54.19 ± 8.23	52.34 ± 7.94	.08
Age at onset	48.70 ± 9.36	—	NA
Duration of illness	5.08 ± 3.11	—	NA
MMSE	27.22 ± 2.51	—	NA
UPDRS III (OFF score) *	34.68 ± 8.80	—	NA
LEDD	598.39 ± 238.50	—	NA

Abbreviations: F: Female; LEDD: Levodopa equivalent daily dose; M: Male; MMSE: Mini mental status examination; UPDRS: Unified Parkinson's disease rating scale, *: UPDRS III (OFF score) was available for 110 subjects.

### 3.2 Atlas construction and quantitative validation

**Figure 2:**
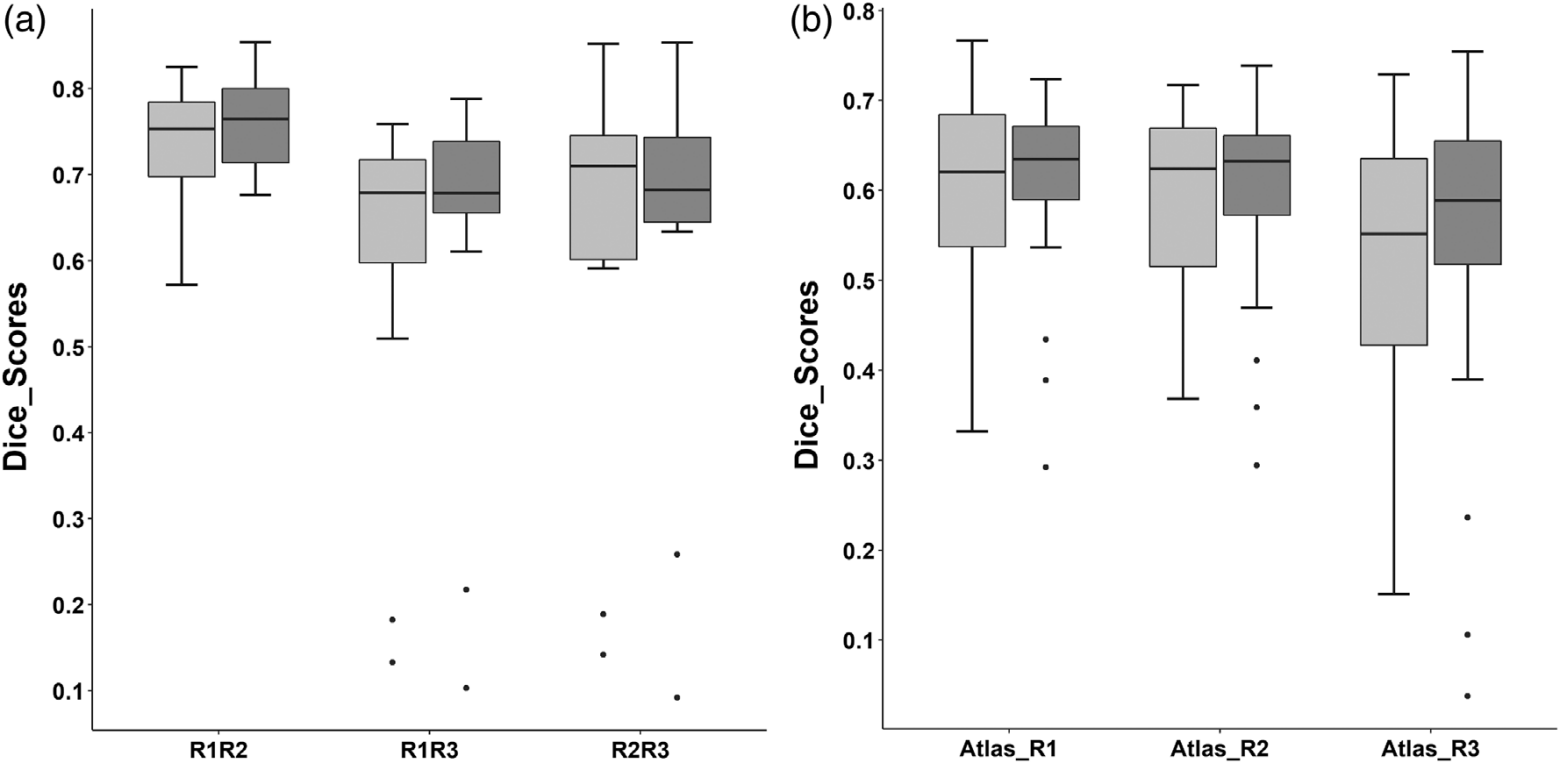
(a) Boxplot of dice coefficient scores amongst raters (b) Boxplot of dice coefficient scores between SNc atlas in subject space and all three raters. Light gray indicates left SNc and dark gray indicates right SNc

The probabilistic and thresholded (0.5) atlas of the SNc computed from NMS‐MRI images of 27 controls is shown in Figure 1. A box plot of dice coefficient scores signifying inter‐rater variability and validation of atlas in shown in Figure 2a,b respectively. The average dice coefficient scores for snROIs amongst raters were as follows ‐R1R2 (left: 0.739 ± 0.066; right: 0.761 ± 0.050), R1R3 (left: 0.601 ± 0.191;right: 0.631 ± 0.199), and R2R3 (left: 0.636 ± 0.205; right: 0.644 ± 0.203). The average dice coefficient for atlas vs. raters were as follows: ATLAS_R1 (left: 0.60 ± 0.106; right: 0.61 ± 0.101), ATLAS_R2 (left: 0.58 ± 0.105; right: 0.6 ± 0.109), and ATLAS_R3 (left: 0.53 ± 0.163; right: 0.56 ± 0.181) as is displayed in Figure 2b. No significant difference (left_snROI: *t* = 1.342, *p*‐value = .196; right_snROI: *t* = 1.247, *p*‐value = .310) was found between overlap of rater's SNc marking and overlap of atlas and rater's markings, thus providing additional support to the reproducibility and validity of the atlas.

### 3.3 Diffusion changes in SNc between PD and HC group

**Figure 3:**
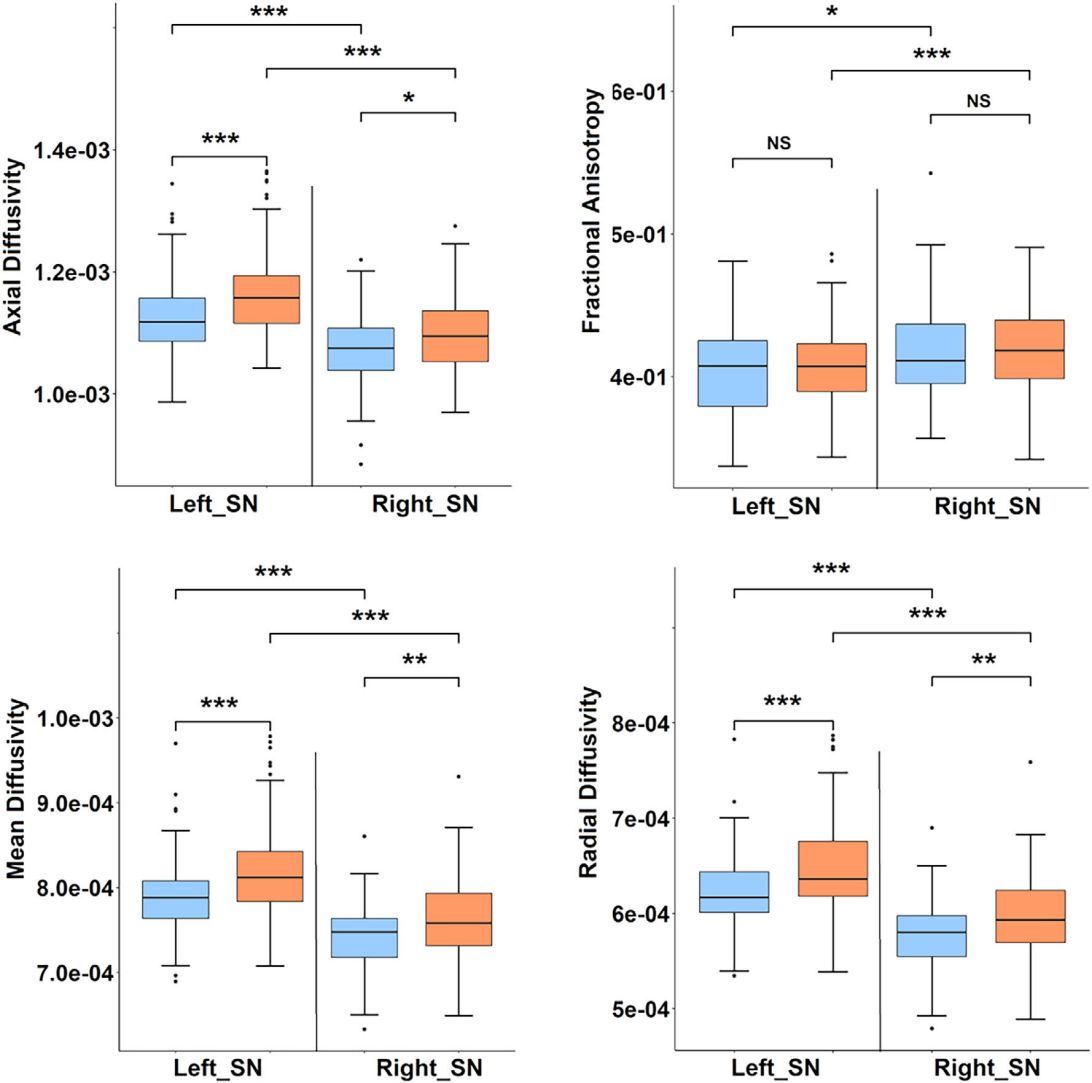
Results of independent *t* test indicating differences in diffusion measures of bilateral substantia nigra in Parkinson's patient and healthy controls. Blue boxplot indicates healthy controls group whereas orange indicates PD patients group. NS: not significant, * *p* < .05, ** *p* < .005, *** *p* < .0005

MANCOVA results showed significantly higher AD (left snROI: *f* = 17.28, *p* = .00004; right snROI: *f* = 6.470, *p* = .011), MD (left snROI: *f* = 21.56, *p* = .000006; right snROI: *f* = 11.15, *p* = .00098), RD (left snROI: *f* = 19.48, *p* = .000016; right snROI: *f* = 11.36, *p* = .00088) in SNc of PD patients as compared to controls. As shown is Figure 3a, FA showed no significant differences (left snROI: *f* = 0.621, *p* = .431: right snROI: *f* = .470, *p* = .493). The mean and *SD* of all diffusion measures in HC and PD groups along with their t‐stats and FDR corrected *p*‐values are shown in Table 3. No significant correlations were obtained between clinical scores and diffusion measures; however, a correlation trend was observed between UPDRS and FA, duration of illness and FA, MD, RD and between FA and age of onset of disease as shown in supplementary Figure S1.

**Table 3:** FDR corrected (*p*‐value = .05) results of independent *t* test for diffusion measures between healthy controls and patients with Parkinson's disease

	PD	HC	*p*‐value	*p*‐value	*p*‐value
Variable	(mean ± *SD*)	(mean ± *SD*)	(PD vs HC)	(PD_L_ vs PD_R_)	(HC_L_ vs HC_R_)
AD_L_	1.17 × 10^−3^ ± 6.94 × 10^−5^	1.13 × 10^−3^ ± 6.76 × 10^−5^	.00002	4.67e‐16	1.43e‐07
AD_R_	1.1 × 10^−3^ ± 5.76 × 10^−5^	1.08 × 10^−3^ ± 5.93 × 10^−5^	.0178	—	—
FA_L_	4.06 × 10^−1^ ± 2.59 × 10^−2^	4.03 × 10^−1^ ± 3.07 × 10^−2^	.5149	1.24e‐04	4.13e‐03
FA_R_	4.19 × 10^−1^ ± 2.96 × 10^−2^	4.17 × 10^−1^ ± 3.35 × 10^−2^	.5149	—	—
MD_L_	8.21 × 10^−4^ ± 5.12 × 10^−5^	7.89 × 10^−4^ ± 4.48 × 10^−5^	.0000	2.32e‐19	1.57e‐13
MD_R_	7.63 × 10^−4^ ± 4.45 × 10^−5^	7.42 × 10^−4^ ± 3.64 × 10^−5^	.0002	—	—
RD_L_	6.49 × 10^−4^ ± 4.72 × 10^−5^	6.21 × ^−4^ ± 3.91 × 10^−5^	.0000	5.48e‐19	4.68e‐15
RD_R_	5.95 × 10^−4^ ± 4.27 × 10^−5^	5.76 × ^−4^ ± 3.36 × 10^−5^	.0002	—	—

Abbreviations: AD: Axial diffusivity; AD_L_: AD of left SNc, AD_R_: AD of right SNc; FA: Fractional anisotropy; FA_L_: FA of left SNc; FA_R_: FA of right SNc; HC: Healthy controls; HC_L_: Left SNc of HC; HC_R_: Right SNc of HC; MD: Mean diffusivity; MD_L_: MD of left SNc; MD_R_: MD of right SNc; PD: Parkinson's disease; PD_L_: Left SNc of patients with PD, PD_R_: Right SNc of patients with PD; RD: Radial diffusivity; RD_L_: RD of left SNc, RD_R_: RD of right SNc; SD: Standard deviation; SNc: Substantia nigra pars compacta.

### 3.4 Asymmetry in diffusion measures of SNc

Asymmetry of all diffusion measures of bilateral SNc was observed for both HC and PD group, with PD group showing a higher significance as shown in Table 3. Left SNc was found to have higher AD, MD and RD values and lower FA values in both groups. Left SNc showed higher AD (HC: *t* = 5.521, *p* = 1.07E‐07; PD: *t* = 8.725, *p* = 3.50E‐16), MD (HC: *t* = 8.075, *p* = 7.87E‐14; PD: *t* = 9.950, *p* = 5.81E‐20), RD (HC: *t* = 8.739, *p* = 1.17E15; PD: *t* = 9.728, 2.74E‐19) as compared to right SNc. FA of the left SNc was found to be significantly lower as compared to right FA in the PD group (HC: *t* = −2.903, *p* = 4.13E‐03; PD: *t* = −3.898, *p* = 1.24E‐04) (HC: *t* = −2.769, *p* = 7.98E‐05; PD: *t* = −4.007, *p* = 7.98E‐05).

### 3.5 Classification of PD and HC based on diffusion measures of SNc

**Figure 4:**
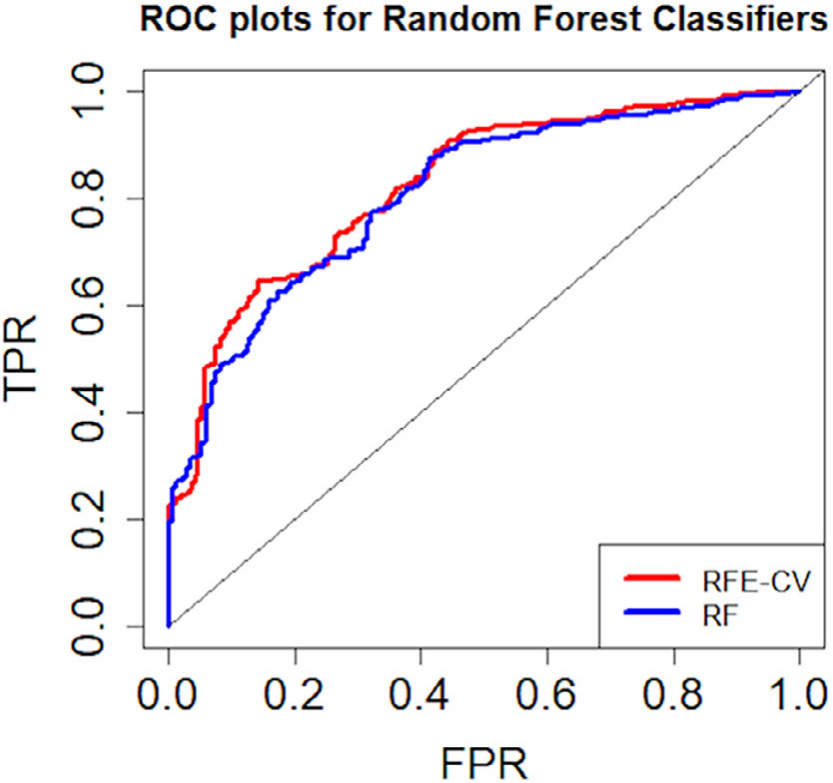
ROC plots for random forest classifiers

The average classification accuracy, sensitivity and specificity for basic RF model were 73.4%, 0.736 ± 0.01, and 0.731 ± 0.01, respectively, whereas that for RF‐RFE model was 71.7%, 0.736 ± 0.01, and 0.686 ± 0.05, respectively. Average feature ranking was consistent for basic RF and RF‐RFE model wherein MD_L_, RD_R_ and RD_L_ were found to be the three topmost ranked features as shown in Table S1. ROC plots indicating average sensitivity and specificity performance of both classifier models is shown in Figure 4.

## 4 DISCUSSION

We created a probabilistic atlas of the SNc by precisely extracting the SNc ROIs using NM rich MR sequence and employed it to accurately delineate the SNc to create a normative atlas that can be used in future PD studies. We applied this atlas to a large cohort of PD patients to gain understanding of the microstructural abnormalities. Our results not only endorsed earlier findings but also facilitated fresh evidence supporting presence of micro‐structural changes in PD substantia nigra compacta using diffusion MRI analysis. We demonstrated higher diffusivity values in the SNc in PD, with no changes in anisotropy and significant asymmetry of the diffusivity values.

The SN is anatomically divided into the SNc and the SN pars reticulata (SNr), where SNc is further subdivided into nigrosomes and the nigral matrix. Nigrosomes are the primary sub regions of the SNc where dopaminergic neurons are degenerated in PD (Blazejewska et al., 2013; Takahashi et al., 2018). The largest of these, nigrosome 1, is positioned in the lateral SN, and is most affected in PD. As described by Takahashi et al, the nigrosome is clearly a part of SNc and hence it is visualized on NMS‐MRI sequence. TheT2 weighted images capture the elevated levels of iron mainly in SNr (Du et al., 2011; Langley et al., 2015; Langley et al., 2016; Langley, Huddleston, Sedlacik, Boelmans, & Hu, 2017). A study by Langley et al. demonstrated that T2‐weighted and NMS‐MRI are sensitive to different sub regions of SN (Langley et al., 2016), and the hypo‐intensity observed on T2 images is unreliable in localizing SNc (Deng et al., 2018; Langley et al., 2016; Wypijewska et al., 2010). A recent study also demonstrated that increase in T2* weighted hypo intense signal is an indication of increase in iron deposition related to PD pathology (Langley et al., 2017). On similar lines, work by Visser et al. employed the FLASH sequence on 7 T MRI to delineate the substantia nigra. However, this sequence does not capture the SNc, as it is sensitive only to the elevated concentrations of ferritin that are prominently observed in SNr (Visser et al., 2016). Therefore, applying the SNr atlas to PD is inappropriate in understanding the abnormalities, which occur predominantly in the SNc owing to the dopaminergic neuronal loss in PD.

To alleviate these limitations, NMS‐MRI has been employed to visualize and quantify the intensity contrast in SNc (Isaias et al., 2016; Matsuura et al., 2013; Matsuura et al., 2016; Ohtsuka et al., 2013; Ohtsuka et al., 2014; Reimao et al., 2015; Sasaki et al., 2006; Schwarz et al., 2011). Earlier studies on NMS MRI (Kitao et al., 2013; Sasaki et al., 2006), through post mortem analysis, have already demonstrated correlation between localization of SNc region from NMS MRI contrast and the histologically delineated SN*c*. Additionally, comparative study on SNc contrast sequences has shown that higher concentration of neuromelanin in SNc is captured by NMS‐MRI (Langley et al., 2015). Moreover, these studies have corroborated its utility not only to render the SNc region but also as a volume or contrast ratio‐based biomarker in PD (Isaias et al., 2016; Kashihara et al., 2011; Matsuura et al., 2013; Matsuura et al., 2016; Ogisu et al., 2013; Ohtsuka et al., 2014; Reimao et al., 2015; Sasaki et al., 2006; Schwarz et al., 2011). However, the techniques employed to extract and analyze the SNc are manual and time‐consuming with low reproducibility. Our work alleviated these issues by creating a SNc template which in future studies would be crucial to overcome the discrepancies in SNc localization by providing a normative baseline for comparison of results across studies.

Our atlas creation was based on uniform and accurate image registration of all subjects to the MNI space. A review study on 14 different nonlinear registration algorithms found that ART and SyN algorithms have consistently performed well across multiple datasets (Klein et al., 2009). We employed a symmetric diffeomorphic (SyN) registration using the ANTs toolbox for registering subject T1 images and subsequently the SNc masks, created from NM rich sequences of 27 subjects onto the MNI space as shown in Figure 1. The maximized optimization of space–time deformation maps in SyN and hierarchical interpolation performed in ANTs, increased normalization accuracy and preserved the brain topology, thus enhancing the registration precision of our probabilistic atlas (Avants et al., 2008; Klein et al., 2009). Each of the registrations was manually checked for precision in registration. The probabilistic atlas created, was thresholded at 50% probability, as it removed the voxels outside the expected SNc region as shown in Figure 1. A consistent dice coefficient score between SNc atlas in subject space and SNc markings by each rater provided a quantitative validation and reproducibility to the atlas.

Our analysis of diffusion measures was performed on a large cohort of patients with PD where we observed a significantly increased MD, RD and AD in the patients compared to age and gender matched healthy controls (Figure 3), with no significant differences in FA. Earlier studies have reported mixed results for significant differences in FA as shown in Table 1. Study on large cohorts by Schuff et al and a few others did not report significant changes in FA (Aquino et al., 2014; Gattellaro et al., 2009; Menke et al., 2010). Our results corroborate these findings, albeit on a completely different dataset. The significant changes were obtained only in the diffusivity measures (MD, RD, AD) as shown in Figure 2b) and were also reflected in the classifier results where the left MD and RD and right RD were captured as the most discriminative features of PD with an accuracy of 71.7% (RF‐RFE).

Degree of myelination, axonal diameter and distance between extracellular membranes drive the changes in radial diffusivity, whereas diffusion anisotropy implies a directional alignment of white matter tracts (Beaulieu, 2002). Intuitively, the biological process of fiber disintegration and de‐myelination which are associated with neurodegeneration, should lead to an increase in RD and reduction in FA values. However, neurodegeneration may involve multiple additional pathological processes such as changes in membrane permeability, restructuring of white matter fibers, glial alterations and damage to the intracellular compartment. The degree of variation in these processes may be contributing towards the proportional changes in diffusion tensors in all three dimensions, and thereby reducing the sensitivity of FA (Acosta‐Cabronero, Williams, Pengas, & Nestor, 2010). Nevertheless, it is important to note that our diffusion protocol was limited to 15 gradient directions which may not facilitate the best model (Jones, Knosche, & Turner, 2013) for fiber tractography or connectivity, but is valid for computing diffusivity and anisotropy measures.

In concurrence with the clinical asymmetry typically reported in PD, which is implicated to an asymmetrical degeneration of dopaminergic nigral neurons, we observed significant asymmetry of diffusion measures in SNc. Although the FA was not significantly different between PD and controls, in patients with PD, we observed that the FA in left SNc was significantly lower when compared to the FA in right SNc (Figure 3). Similarly, MD, AD and RD also demonstrated significantly higher values in the left SNc when compared to right (Figure 3). We did not have details pertaining to clinical laterality, owing to which we were unable to ascertain the concordance between clinical lateralization and diffusion asymmetry. However, earlier work by our group has illustrated the correlation of clinical asymmetry and laterality with the asymmetry of the SNc using contrast ratios on NMS‐MRI (Prasad, Saini, Yadav, & Pal, 2018).

Our correlation analysis did not demonstrate any significant associations of DTI measures with AoO, DoI, UPDRS‐III OFF, or LEDD scores. However, a trend was observed between UPDRS, and FA, DoI and FA, MD, RD and between AoI and FA (Supplementary Figure S1).

## 5 CONCLUSIONS

In conclusion, this study addressed a crucial question of uniform SNc localization in patients with PD and performed a large‐scale robust assessment of microstructural features of the SNc in PD using diffusion measures. Our standardized SNc atlas based on the NMS‐MRI will be released to the scientific community and will further aid in eliminating the methodological variability associated with delineation of SNc. Microstructural abnormalities of the SNc in PD are predominantly associated with altered diffusion metrics rather than anisotropy, and demonstrate a significant asymmetry which is in concurrence with clinical lateralization of symptoms. These results obtained from our large‐scale study on an accurately delineated SNc provide a thorough and reliable profile of the neurodegeneration associated microstructural abnormalities of the SNc in PD.

## Supporting information


**Figure S1** Correlations between DTI measures and clinical scores which showed borderline significance.
**Figure S2:** Scatter plot of diffusion measures in left and right SN for PD and HC group.
**Table S1:** Average feature rankings obtained from 10 repetitions of RF and RF‐RFE models.
**SNc atlas link:** https://github.com/apoorvasafai/NMS-SNc-atlas (Soon to be uploaded on NITRC forum)

## Data Availability

Anonymized data and code used in this manuscript will be shared at the request of qualified investigators. The SNc atlas constructed in this study is made freely available on github website.
