# Supplementary material for: Microstructural abnormalities of substantia nigra in Parkinson's disease: A neuromelanin sensitive MRI atlas based study
Source: Hum Brain Mapp. 2019 Nov 28;41(5):1323–33. doi: 10.1002/hbm.24878 (PMC7267920; doi:10.1002/hbm.24878)
Supplement: Supplementary file 1 — Figure S1 Correlations between DTI measures and clinical scores which showed borderline significance. Figure S2: Scatter plot of diffusion measures in left and right SN for PD and HC group. Table S1: Average feature rankings obtained from 10 repetitions of RF and RF‐RFE models. SNc atlas link: https://github.com/apoorvasafai/NMS-SNc-atlas (Soon to be uploaded on NITRC forum) [file HBM-41-1323-s001.docx]

**Correlation Analysis:**

Pearson’s correlation was performed on all DTI measures and age and gender regressed, residual clinical scores such as UPDRS III (off), Age of Onset (AoI),Duration of Illness (DoI) and Levodopa Equivalent Daily Dosage (LEDD). Although no signficant correlations were obtained, a trend was observed between diffusion measures of left SNc and clinical scores. FA of left SNc showed a negative correlation trend with DoI (r=-0.146, p=0.114) and UPDRS scores (r=-0.184, p=0.056) and a positive trend with AoI (r=0.138,=0.113). MD (r=0.105, p=0.256) and RD (r=0.131, p=0.157) both showed a positive correlation trend with DoI.


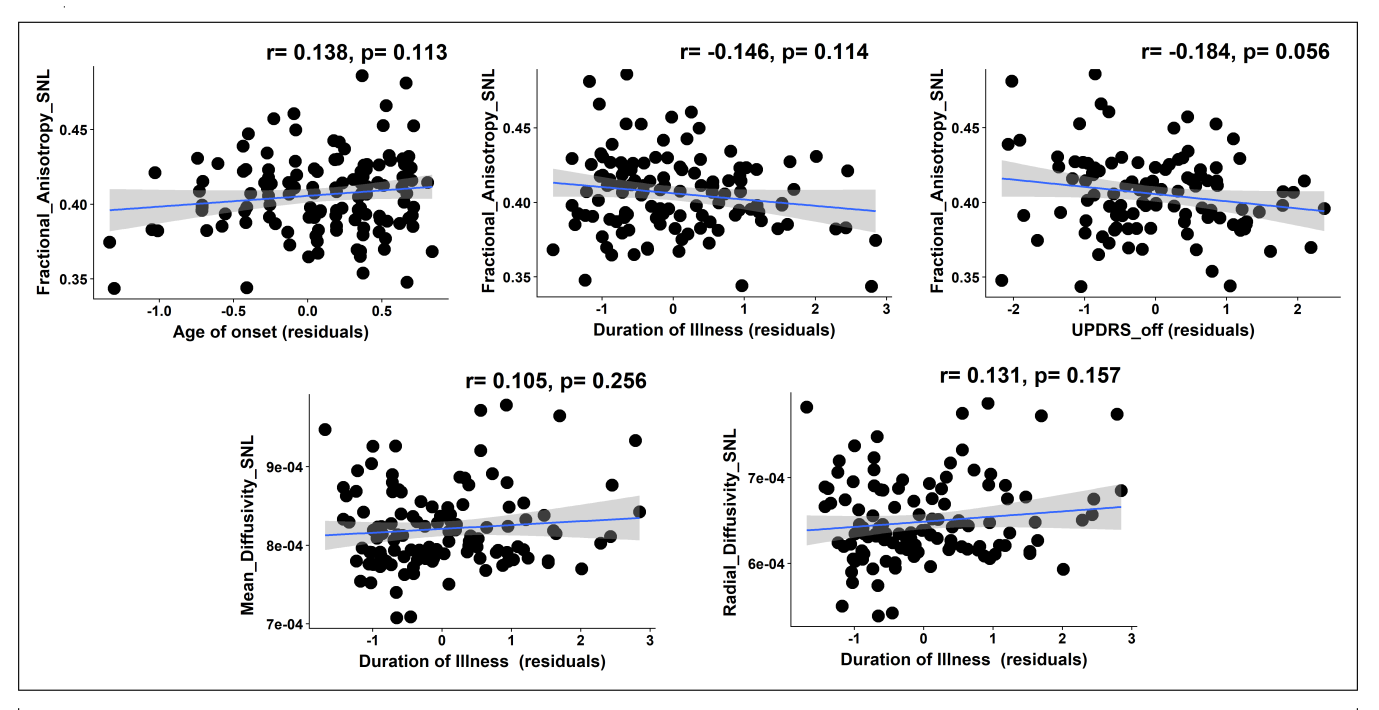


**Figure-S1.** Correlations between DTI measures of left SNc and residual clinical scores with age and gender regressed


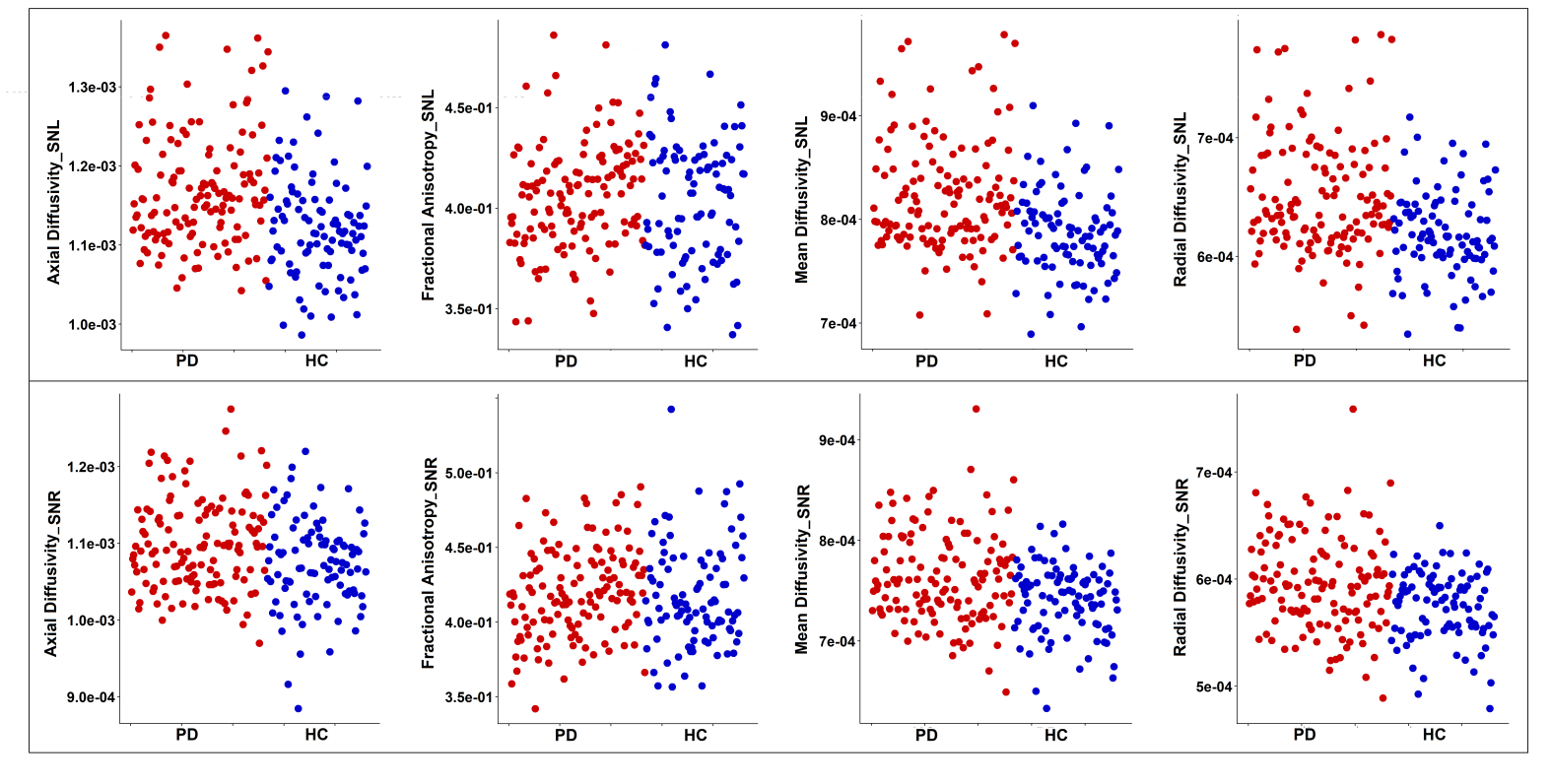
**Figure-S2.** Scatter plot of diffusion measures in left and right SN for PD and HC group.

**Table-S1.**Average feature rankings obtained from 10 repetitions of RF and RF-RFE models.

| Features | Feature Rankings | |
| --- | --- | --- |
|  | RF | RF-RFE |
| AD_L_ | 7.6 | 7.6 |
| FA_L_ | 5.3 | 5.3 |
| MD_L_ | 1 | 1 |
| RD_L_ | 2.7 | 2.8 |
| AD_R_ | 7.4 | 7.4 |
| FA_R_ | 4 | 4 |
| MD_R_ | 5.7 | 5.7 |
| RD_R_ | 2.3 | 2.2 |

(AD: Axial diffusivity; AD_L:_ AD of left SNc, AD_R:_ AD of right SNc; FA: Fractional anisotropy; FA_L:_ FA of left SNc; FA_R:_ FA of right SNc; HC: Healthy controls; HC_L_: Left SNc of HC; HC_R_: Right SNc of HC; MD: Mean diffusivity; MD_L:_ MD of left SNc; MD_R:_ MD of right SNc; PD: Parkinson’s disease; PD_L_: Left SNc of patients with PD, PD_R_: Right SNc of patients with PD; RD: Radial diffusivity; RD_L:_ RD of left SNc, RD_R:_ RD of right SNc )
